# Awake Tracheal Intubation Training in a Newly Established Anaesthesia Department: An Audit of Over 200 Patients With Anticipated Difficult Airway

**DOI:** 10.7759/cureus.92513

**Published:** 2025-09-17

**Authors:** G Niraj, Sukanya Biradar, Jyothsna Singh, Harshitha Naidu, Gurudatt Shenoy

**Affiliations:** 1 Pain Medicine and Anaesthesia, Sri Madhusudan Sai Institute of Medical Sciences and Research, Chikkaballapur, IND; 2 Anaesthesia, Sri Madhusudan Sai Institute of Medical Sciences and Research, Chikkaballapur, IND

**Keywords:** awake tracheal intubation, difficult airway, physiological difficult airway, posttraumatic stress disorder, spray as you go, transtracheal injection

## Abstract

Background: Awake tracheal intubation (ATI) remains the gold standard in the management of a difficult airway. However, training in this technique is suboptimal. This has major implications for patient safety as well as anaesthetists’ skill set. The report presents a quality improvement project on ATI at a newly established tertiary care hospital in India.

Methods: This project included an initial audit to identify the gaps in knowledge and skill in ATI, implementation of identified measures, followed by a re-audit to evaluate competency in ATI and patient satisfaction with airway management. Patients were followed up over the telephone at 12 weeks post-procedure to assess recall of the event. The primary outcomes included patient satisfaction with the management of difficult airway and any adverse recall of events at three-month follow-up.

Results: The initial audit was performed in April 2024, which revealed a paucity of skill in ATI, the unavailability of flexible bronchoscopes for training, and a lack of a formal protocol. The re-audit was performed from May 2024 to April 2025. During this period, 203 ATIs were performed by 16 anaesthetists with a 100% success rate. Patient satisfaction with pre-procedure counselling and airway management was high. The complication rate was low. At the 12-week telephone review, 45% of patients recalled the event, and 9% of patients had a negative recall associated with the event.

Conclusion: Training in ATI should be integrated with routine airway management to enhance expertise and confidence in the technique as well as patient safety.

## Introduction

Awake tracheal intubation (ATI) is defined as the successful placement of a tracheal tube in a patient who is awake and breathing spontaneously. Although ATI is the gold standard for the management of the anticipated difficult airway, it is an underused technique [1]. Despite its high success rates and excellent risk profile, training in ATI remains suboptimal [2-4]. The recently published Difficult Airway Society ATI guidelines reiterate that ATI is a core skill [3]. Flexible bronchoscopes with standalone screens have helped enhance the practice of ATI. In addition, the availability of manikins has improved proficiency in flexible scope handling [5]. Evidence suggests that an acceptable level of competence can be achieved after 10 ATIs [6]. However, clinical opportunities for ATI training are limited, which curtails familiarity and expertise in the technique [7]. This mandates prioritisation of ATI as a core skill and innovative training methods to enhance confidence as well as competence in ATI.

This report presents an initial audit evaluating the knowledge and skill of ATI among anaesthetists of a newly established anaesthesia department in a medical college situated in rural south India. Following the audit, measures were implemented to enhance training in ATI, improve the availability of flexible bronchoscopes, and develop a protocol for ATI. Intensive training in ATI was provided to the entire department. Thereafter, a re-audit was performed over a 12-month period to evaluate competence in ATI and patient satisfaction with difficult airway management. This report presents the findings of the initial audit and re-audit of ATI in over 200 patients with anticipated difficult airways.

## Materials and methods

Methods

The objective of the initial audit was to evaluate the current knowledge and skill of performing ATI among consultants and senior residents in a recently established Department of Anaesthesia at Sri Madhusudan Sai Institute of Medical Sciences and Research (SMSIMSR), Chikkaballapur, India.

The benchmarks included knowledge of the indications and technique of ATI; a minimum of 10 ATIs performed per anaesthetist; the ability to perform ATI independently; availability of an ATI protocol; and a fibreoptic bronchoscope. As this was a quality improvement project, ethics board review was not undertaken. The audit was registered with the Clinical and Medical Audit Committee (SMSIMSR/CMAC/17A) and was performed in April 2024 using a questionnaire-based anonymised survey. All members of the anaesthetic team completed the paper-based survey (Appendix A). Following the analysis of the survey, measures were taken to enhance the practice of ATI in the department. These included acquiring a flexible bronchoscope, protocol with a checklist for ATI, training on an airway manikin to enhance flexible scope handling, training on identification of an anticipated difficult airway at pre-anaesthetic evaluation, and hands-on training in ATI (Appendix B).

Training in identifying difficult airways for ATI

During comprehensive airway assessment in a stand-alone pre-anaesthetic clinic, patients with two or more factors predictive of an anticipated difficult airway were scheduled to undergo ATI via the oral route. These factors included a short thick neck, modified Mallampati Class 3 or 4, and mouth opening <3 finger breadth, buck teeth, retrognathia, body mass index >35, history of snoring, obstructive sleep apnoea, history of difficult intubation or mask ventilation, and limited range of motion at the cervical spine [8].

Training in ATI

Initial training in flexible bronchoscope use was performed on an airway manikin (AMBU® Airway Man, AMBU, Denmark). The training in ATI via the oral route was provided by a skilled departmental airway lead with experience in performing over 200 ATIs. In addition to flexible scope handling, training in two techniques for achieving anaesthesia of the subglottic area was provided (transtracheal injection and spray-as-you-go). The entire anaesthetic team underwent training and was evaluated.

Informed consent

The patients were informed of the plan for ATI at preassessment. This was followed by a detailed discussion on the rationale for ATI and the technique. All patients provided an informed written patient consent for ATI. Additional consent was taken for telephone review at three months after surgery, as well as for the publication of de-identified data in a peer-reviewed journal.

Re-audit

Subsequently, a re-audit was performed over a 12-month period to assess competence in ATI via the oral route, the informed consent process, adherence to the ATI protocol, patient satisfaction with difficult airway management (Appendix C), and to detail complications (desaturation with SpO_2_ < 90%, more than three attempts, airway trauma) as well as failure of ATI.

The outcomes measured included operator grade, time taken for ATI, number of attempts at ATI, number of operators, local anaesthetic technique for subglottic anaesthesia (transtracheal injection or spray-as-you-go), patient satisfaction with airway management, and any complication (desaturation with SpO_2_ < 90%, airway trauma, sore throat over three days, oesophageal intubation). Patients were reviewed by telephone at 12 weeks following hospital discharge to assess the presence of any negative recall of the procedure (Appendix D).

Statistical analysis

Descriptive statistics are presented as frequency for categorical variables and median with interquartile range (IQR) for continuous variables.

## Results

Initial audit

The newly established department consisted of 15 anaesthetists, including two professors, one additional professor, four assistant professors, six senior residents, and two first-year anaesthetic trainees (junior residents). The initial audit evaluating knowledge and skill was performed over a two-week period in April 2024. Only 13 members who had completed training in anaesthesia were audited.

The results of the audit revealed that there was adequate knowledge of the technique and indication for ATI. All audited members correctly answered the questions on the indication for ATI (13/13, 100%). Eight anaesthetists (8/13, 62%) were able to detail the ATI technique. However, only one anaesthetist had performed a minimum of 10 ATIs and was able to perform ATIs independently (1/13 = 8%). Although a fiberoptic bronchoscope (Olympus BF-PE2, Olympus Medical Systems, India) was available, the screen (to connect the bronchoscope) was not easily available, as it was shared by various surgical departments. In addition, the high-end fiberoptic bronchoscope was not suitable for training purposes. In addition, a formal ATI protocol was unavailable.

Re-audit

Following implementation of the steps mentioned in the Methods section, including a formal ATI protocol, the re-audit was performed over a 12-month period (01 May 2024 to 30 April 2025). During the re-audit period, the department had grown to 16 anaesthetists. The new anaesthetists were included in the re-audit.

The results revealed that there was significant improvement in the knowledge of airway assessment, identification of anticipated difficult airway, and competence in the ATI technique. During the 12-month audit period, a total of 6,618 surgeries were performed that included 2,080 general anaesthesia cases. During the period, a total of 203 ATIs were performed. Informed written consent was obtained from all patients (100%). All 16 anaesthetists, including the two first-year trainees, had performed a minimum of 12 ATIs each. At the end of the 12-month period, all 16 anaesthetists in the department, including the two junior residents, were competent to perform ATI independently. Since the start of the re-audit, there has been no instance of rescheduling surgery for patients with a difficult airway due to the lack of a trained ATI operator. Technical details are provided in Table 1.

**Table 1:** Demographic data and awake tracheal intubation technical details SD: standard deviation; IQR: interquartile range

	N = 203
Age, mean (SD)	49.6 (16)
Gender (M: F)	111: 92
Airway difficulty factors	N (%)
2	129 (64)
3	54 (26)
4	20 (10)
Anticipated airway identified	N (%)
Pre-anaesthetic clinic	196 (97)
Pre-anaesthetic bay	7 (3)
Number of lidocaine sprays, median (IQR)	8 (8, 8)
Subglottic anaesthesia	N (%)
Trans-tracheal injection	198 (98)
Spray-as-you-go	5 (2)

ATI technique

All patients received an intravenous mixture of 1 mg of midazolam, 4 mg of ondansetron, and 200 mcg of glycopyrrolate as premedication on arrival to the operating room. Fentanyl (30-50 mcg) was used for providing conscious sedation as remifentanil was unavailable. Supplemental oxygen (2 L) was provided by nasal cannula. A second anaesthetist was present during ATI. Topical anaesthesia was achieved with 10% lidocaine spray to the oral and oropharyngeal cavity (8-10 sprays). Sub-glottic anaesthesia was achieved by trans-tracheal injection of 4 mL of 2% lidocaine or spray-as-you-go technique with 4 mL of 2% lidocaine. Once the trachea was intubated, correct placement was confirmed by end tidal carbon dioxide tracing. After confirmation of correct tube placement, general anaesthesia was induced with 100 mg propofol and 30 mg of atracurium. Anaesthesia was maintained with oxygen, air, and an isoflurane mixture.

The intensive training in ATI also enhanced training for operating theatre technicians. Only two technicians (2/10, 20%) had assisted with ATI prior to the audit. At the end of the 12-month period, all 10 technicians achieved competence in assisting the operator during ATI.

During the re-audit period, the incidence of unanticipated difficult airway recorded in the department was 1% (21/2,080). The number of unanticipated difficult airways that required waking up the patient and subsequent ATI was 0.1% (2/2,080). There were no cases of ATI failure during the re-audit period.

Major complications recorded during the re-audit included desaturation (6/203, 3%), oesophageal intubation during railroading of the endotracheal tube (2/203, 1%), and reintubation of the trachea post-extubation due to persistent desaturation (1/203, 0.5%). Oesophageal intubation was promptly identified, and ATI was performed successfully. Minor complications included a persistent sore throat over three days (16/203, 8%).

Patient satisfaction

Almost all patients were satisfied with pre-procedure counselling for ATI (197/203, 97%). Two patients were unsatisfied (2/203, 1%) while data was unavailable for four patients (4/203, 2%). Patient satisfaction with difficult airway management was excellent (12/203, 6%), good (170/203, 84%), and fair (21/203, 10%).

Twelve-week telephone review

The 12-week telephone review was completed with 171 patients (171/203, 84%). Ninety-six patients (96/171, 56%) reported having no recall of the event, while 75 patients (75/171, 44%) reported successful recollection of ATI. Fifteen patients (15/171, 9%) reported negative recall of the event. These included persistent sore throat (9/171, 5%), voice change for a few days (3/171, 2%), and an unpleasant, persistent memory (3/171, 2%). The three patients with unpleasant, persisting memories were offered a referral to the clinical psychology department for further management.

## Discussion

We present the findings of a quality improvement project assessing the knowledge, skill, and training of ATI at a newly established anaesthetic department based in a tertiary medical college located in rural India. Following gap analysis and implementation of remedial measures, all the anaesthetists, including very junior trainees, attained competency in performing ATI independently within a 12-month period. To the best of our knowledge, this is the first report where an entire anaesthetic department achieved competence in ATI. In addition, patients who underwent ATI were prospectively reviewed at 12 weeks to evaluate for any negative experience.

ATI is recommended when difficulty is anticipated with airway management [3]. Poorly performed ATI can be traumatic to the patient and can impact the whole team. It is widely accepted that there is deskilling in this core skill [3,4]. Potential reasons include perceived patient discomfort, lack of formal training, widespread use of supraglottic devices, availability of a video laryngoscope (VLS), insufficient clinician experience, poor judgment, and the availability of flexible bronchoscopes [9,10]. Law et al. reported on the use of ATI at a tertiary centre and found that ATI utilisation decreased by 50% over the seven-year study period [11].

Due to the widespread availability of VLS and supraglottic airway, there is greater reliance on these technologies. As a result, there is a high threshold to trigger ATI. All anaesthetists require advanced airway skills to enable safe management of airway difficulties and crises. These skills need to be learnt and frequently updated [12]. ATI has been duly recognised as a core skill [3]. It is reported that competence can be achieved by performing 10 ATIs [5]. The only absolute contraindication for ATI is patient refusal [9]. The ability to secure the airway of a patient who maintains their intrinsic airway tone underpins the superior safety profile of ATI over techniques with the patient deeply sedated or anaesthetised [3].

Since the inception of the department in January 2023 until April 2024, a total of 16 ATIs were performed using the Olympus fibrescope by two operators. In the remaining cases of anticipated difficult airway, a VLS was utilised. In April 2024, the VLS malfunctioned, and there was only one operator available to perform ATI independently. When the operator was unavailable, surgery on patients with difficult airways had to be rescheduled. This necessitated the initial audit and subsequent ATI training. ATI accounted for <1% of all airway interventions prior to this audit. This is in line with published literature [13]. During the 12-month period of the re-audit, ATI constituted 9.8% (203/2,080) of general anaesthesia cases with a success rate of 100%. The incidence of anticipated difficult airway in our cohort is in concordance with published literature [9,14]. In addition, the major complication rate was low at 4.4% (9/203). The median (IQR) time to perform ATI was four minutes (IQR range 3-5 minutes). Patient satisfaction with pre-procedure counselling as well as with the ATI technique was high. We aimed to demonstrate that integrating ATI into routine airway management can vastly enhance patient care, clinician expertise in ATI, and is an achievable exercise [15].

ATI can be associated with the significant operator-related stress of all elective airway management interventions [16]. These stressors may be associated with suboptimal performance, increasing the risk of complications, including failure [17,18]. It is necessary to develop an ATI protocol, which includes a checklist that serves as a cognitive aid as well as streamlines the technique to achieve initial competency in ATI. This is important as complication rates are often related to operator experience irrespective of training grade [19]. We developed an ATI protocol that facilitated rapid expertise in the technique with minimal adverse outcomes and manageable procedural time. Since May 2024, not a single case of anticipated difficult airway has been rescheduled due to a lack of ATI expertise. In addition, nine ATIs (9/203, 4.5%) were performed in physiologically difficult airways (PDAs). These included patients with a full stomach for emergency surgery (7/9, 77%) and haemodynamic instability (2/9, 23%). We are in concordance with the recommendation by Gómez-Ríos et al. that PDA should be considered an indication for ATI [20].

Limitations of the study include it being a single-centre report from a newly established anaesthetic department. However, the model can be easily replicated in larger units and will go a long way in re-establishing ATI as the gold standard technique in patients with anticipated difficult airways. In addition, it will reinforce thorough assessment of the airway and enhance competency in ATI. Training for ATI was predominantly focused on oral ATI as the technique requires greater skill in comparison to nasal ATI [21]. Identification of the cricothyroid membrane is key in establishing front-of-neck access. Training in transtracheal injection of local anaesthetic can enhance identification of the cricothyroid membrane during airway crises. Despite adequate preoperative counselling, sedation, and assiduous technique, three patients (3/203, 1.48%) had developed features suggestive of posttraumatic stress disorder (PTSD) at the 12-week review. Intraoperative awareness was ruled out. They were offered psychological therapy.

## Conclusions

In conclusion, the authors recommend that training in ATI should be integrated with routine airway management. This will enhance expertise and confidence in the technique as well as patient safety. Integration as a part of routine airway management will arrest this downward trend in the practice of ATI. The audit resulted in all anaesthetists in the department gaining competency to perform ATI independently with minimal adverse effects. Despite extensive counselling and care, three patients developed features suggestive of PTSD at the 12-week review. The authors recommend long-term follow-up of patients who have undergone ATI to rule out psychological distress.

## Appendices

Appendix A

Awake Tracheal Intubation: Knowledge and Experience

Grade (Please tick): Consultant/Senior Resident

Previous experience of awake tracheal intubation (ATI): 

1. Approximately how many times have you performed ATI as the lead anaesthetist?

                          Ever?  …………………………..

                          In the last year? ………………….

 2. Approximately how many times have you observed or assisted with ATI? 

                          Ever? …………….

                          In the last year? …………………….

3. Have you ever received any training in ATI? 

                                    Yes                      No

 4. If yes, specify when you last received any training 

                         • within the last year

                         • 1-2 years 

                         • 3-4 years

                         • 5-10 years

                         • >10 years

 5. If yes, please specify the type of training received 

                        • Mannequin/airway box

                        • volunteer subjects

                        • awake patients

                        • anaesthetised patients 

                        • other (please specify) 

6. Would you feel confident about performing ATI as the sole anaesthetist? 

                         • Yes                                       • No

Appendix B

Awake Tracheal Intubation (Oral Route) Protocol

• Secure an intravenous cannula and attach standard monitoring in the pre-anaesthetic bay area 

• Intravenous ondansetron 4 mg, midazolam 1 mg and glycopyrrolate 200 microgram

• Lidocaine sprays to the lips, oral cavity, and oropharynx = 5-7 sprays. The maximum dose of lidocaine is not to exceed 9 mg.kg-1 lean body weight

• Shift to the operating room and connect supplemental oxygen 2 L via nasal prongs

• Further 3 sprays of lidocaine to the oropharynx

• Presence of a second anaesthetist during awake tracheal intubation (ATI)

• Sedation with fentanyl 30-50 mcg, depending on body weight

• Technique I: Transtracheal injection of 4 ml of 2% lidocaine 

   Technique II: Spray as you go: Spray 2% lidocaine through the port of the flexible scope just above (2 ml) and below the vocal cords (2 mL)

• Check for anaesthesia of the oral and oropharynx by inserting a suction catheter into the oropharynx

• A suitably sized reinforced endotracheal tube (ET) with the bevel of the tracheal tube facing posteriorly 

• Introduce the flexible bronchoscope and manoeuvre it into the trachea

• Check end tidal CO_2_ and visual confirmation of ET placement (two-point check)

• Once end tidal CO_2_ is confirmed,  general anaesthesia is induced with intravenous propofol and atracurium for muscle relaxation

• Intravenous dexamethasone 4 mg following induction of general anaesthesia

• At the end of the surgery, prior to the reversal of muscle relaxation, a suitable-sized nasotracheal tube is inserted following lubrication

• Following reversal of muscle relaxation, the trachea is extubated with the patient sitting up, spontaneously breathing and in the presence of a second anaesthetist

Appendix C

Patient Satisfaction Survey

1. How satisfied were you with the counselling on awake tracheal intubation procedure that you received during pre-anaesthetic check-up?

• Satisfied

• Unsatisfied

2. How satisfied were you with the management of your airway during surgery?

• Excellent

• Good

• Fair

• Poor

Appendix D

Twelve-Week Telephone Review Following Awake Tracheal Intubation

1. Do you recall any event during the management of your airway?

• No

• Yes

2. If yes, do you have any negative recall of the event during the management of your airway?

• No

• Yes

3. If yes, do you have any unpleasant memories of the event during the management of your airway?

•No

•Yes

4. If yes, would you like a referral to the clinical psychology department for an assessment?

• No

• Yes
